# Stability of remission rates in a 3-year follow-up of naturalistic treated depressed inpatients

**DOI:** 10.1186/s12888-016-0851-4

**Published:** 2016-05-20

**Authors:** Florian Seemüller, Michael Obermeier, Rebecca Schennach, Michael Bauer, Mazda Adli, Peter Brieger, Gerd Laux, Michael Riedel, Peter Falkai, Hans-Jürgen Möller

**Affiliations:** Department of Psychiatry and Psychotherapy, Ludwig-Maximilians-University Munich, Nussbaumstrasse 7, 80336 Munich, Germany; Department of Psychiatry and Psychotherapy, Carl Gustav Carus University Dresden, Technical University Dresden, Fetscherstr. 74, 01307 Dresden, Germany; Department of Psychiatry and Psychotherapy, Campus, Charité Mitte (CCM), Charitéplatz 1, 10117 Berlin, Germany; Department of Psychiatry and Psychotherapy, Martin-Luther University Halle-Wittenberg, Julius-Kühn-Str.7, 06097 Halle, Germany; Department of Psychiatry and Psychotherapy, kbo-Inn-Salzach-Klinikum, Gabersee 7, 83512 Wasserburg, Germany; Department of Psychiatry and Psychotherapy, Vinzenz von Paul Hospital, Rottweil, Germany; Department of Psychiatry, Psychosomatic and Psychotherapy, kbo-Lech-Mangfall-Klinik, Garmisch-Patenkirchen, Germany; Department of Psychiatry, Psychosomatic and Psychotherapy, Bezirkskrankenhaus Kempten, Robert-Weixlerstrasse 46, 87435 Kempten, Germany

## Abstract

**Background:**

Remission is a common outcome of short-term trials and the main goal of acute and longterm treatment. The longitudinal stability of remission has rarely been investigated under naturalistic treatment conditions.

**Methods:**

Naturalistic multisite follow-up study. Three-year symptomatic long-term outcome of initially hospitalized tertiary care patients (*N* = 784) with major depressive episodes. Remission rates as well as the switch rates between remission and non-remission were reported.

**Results:**

After one, two and three years 62 %, 59 % and 69 % of the observed patients met criteria for remission. During the follow-up 88 % of all patients achieved remission. 36 % of maintained remission from discharge to 3-years, 12 % of all patients never reached remission and 52 % percent showed a fluctuating course switching from remission to non-remission and vice versa. There was considerable transition between remission and non-remission. For example, from discharge to 1 year, from 1 to 2, and from 2 to 3 years 25 %, 21 % and 11 % lost remission.

**Conclusion:**

Cumulative outcome rates are encouraging. Absolute rates at predefined endpoints as well as the fluctuations between these outcomes reflect the variable and chronic nature of major depression.

## Background

Drug approval authorities like the European Medicines Agency demand that clinical relevant outcome criteria like remission should be used in antidepressant short-term trials [1]. Also in clinical practice remission, commonly defined as the virtual absence of depressive symptoms, is still one of the main goals of acute antidepressant treatment [2, 3]. Evidence from naturalistic long-term studies of depressed inpatients suggests that up to 90 % achieve a full remission over a 2–5 year period [4–8]. In addition, remitted patients experience significantly lower relapse rates [9, 10].

On the other hand, it is known that the nature of depression is rather unstable: Symptoms improve and worsen over time and patients can switch between a symptomatic and a remitted state [10, 11, 12, 13]. However, the stability of remission so far has only rarely been reported. Most long-term reports tend to focus either on cumulative remission rates or on relapse rates, but do not report how many patients develop symptoms again and leave the remitted state after a certain period. Thus, the fluctuations inherent in major depressive disorder can easily be missed and cumulative remission rates might lead to an overestimation of positive outcome in major depression. Moreover, the majority of results on remission relies on data from randomized controlled trials with limited generizability with respect to a real world settings [4–8].

With regards to inpatient treatment our group recently reported that among a representative sample of tertiary care inpatients (*N* = 1014) with major depressive episode more than 50 % of all patients reached remission at discharge [14, 15].

Here we present the 3-year long-term results of this prospective, multicentre follow-up trial on inpatients with major depressive disorder. This cohort was followed up annually after discharge from inpatient treatment with attention to course and outcome of the major depressive disorder. The current report complements the description of the acute inpatient outcome. Relapse rates and risk factors have already been reported in a companion paper [10].

In this report we specifically aimed at investigating the rates and the stability of remission at the 1, 2 and 3 year follow-up. Additionally we sought to investigate how many patients never remit from discharge on and to get an estimate of the antidepressant treatment level during the follow-up.

## Methods

### Study overview and organization

This prospective naturalistic multicenter follow-up was primarily designed to address the issues of treatment resistance, relapse, chronicity and suicidality in depressive disorders within the framework of psychiatric university and district hospitals. It was part of the German research network on depression (GRND) and was funded by the German Federal Ministry of Education and Research (BMBF). The study was planned to be conducted in representative inpatient groups and settings using clinical management tools that easily can be applied in daily practice.

The follow-up consisted of two parts: a) the naturalistic acute inpatient treatment period with biweekly measurements [14], which was followed by b) a long-term naturalistic follow-up lasting up to 3 years after discharge. Here, results of the three year follow-up were presented. Twelve study centers across Germany participated in this trial, including seven university hospitals (Berlin: Campus Charité Mitte and Campus Benjamin-Franklin, Düsseldorf, Halle, Heidelberg, Munich: MPI and LMU) and five district hospitals (Gabersee, Inn-Salzach-Clinic/Bavaria, Haar, Isar-Amper-Clinic/Bavaria, Berlin: Auguste-Viktoria-Hospital, St.-Joseph-Hospital and St.-Hedwig-Hospital). Clinical research coordinators at each site assisted in protocol implementation and computerized data collection.

## Experimental procedures

### Sample and data collection

The diagnose of a depressive spectrum disorder according to DSM-IV was confirmed at baseline and at discharge at the end of the acute phase and at each annual follow-up visit using the Structured Clinical Interview for DSM-IV (SCID-I) [16]. SCID II was applied to carefully assess comorbid axis II personality. To allow inclusion of clinical representative populations the following inclusion and exclusion criteria was applied:

Inclusion criteria were:

1. Age between 18 and 65
2. Signed written informed consent
3. Hospitalization and fulfilling of ICD-10 diagnostic criteria for any major depressive episode (ICD-10:F32, F33, F34, F38) or for a depressive disorder not otherwise specified (ICD-10: F39) as primary diagnosis.

Exclusion criteria were:

1. Organic cause of depression
2. Insufficient knowledge of German language
3. Distance from place of residence to the study center of more than 100 km

The 3-year follow-up period started after discharge from inpatient treatment.

The annual follow-up ratings further included, the Hamilton Depression Rating Scale, (HAMD-17) [17], the MADRS [18] and the collection of socio-demographic and clinical variables using the systematic basic assessment scale of clinical and socio-demographic variables in psychiatry (BADO) [19]. These methods were described in detail in a study protocol, which allowed post-hoc analyses. The respective local Ethics Review Committee of each participating site has approved the study protocol. Signed written informed consent was obtained from all participants.

### Treatment

During the follow-up, patients were treated naturalistically at the discretion of the respective outpatient psychiatrist/neurologist, family doctor or general practitioner in charge. All medication decisions including medication discontinuation and switches were made by the respective physicians. Treatment was recorded as prescribed medication class at each follow-up visit.

### Outcome criteria

HAMD-17 and MADRS total scores were calculated for all follow-up visits. Remission was defined as HAMD-17 total score ≤ 7 [20].

### Statistical analysis

In order to assess if drop-outs were at random, group comparisons between drop-outs after one year and the 3-year follow up sample were conducted using Fisher’s exact test, in case of categorical data and Wilcoxon-Mann-Whitney-U test in case of metrical data, respectively.

In order to account for the comparably high dropout rate we looked at two different samples 1) The LOCF sample with a total number of 784 patients at each visit and 2) The completer sample (=observed case analysis, OC) of 148 patients, with visits at each follow-up. Due to the exploratory character of these group comparisons there is no multiple testing problem and therefore no respective corrections have been performed.

All statistical analyses were performed using the statistical software environment R 2.11.1 [21].

## Results

### Patient disposition

**Fig. 1:**
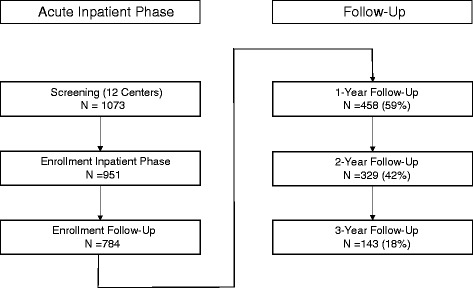
Flow-chart for patients showing the number of available patients

Detailed description of patient disposition of the acute treatment phase can be found elsewhere [14]. The numbers of patients eligible for follow-up analysis at baseline, at 1, 2 and 3 years are displayed in the flow chart in Fig. 1.

Of all (*n* = 1073) enrolled patients 1014 patients had complete baseline HAMD ratings. At discharge 784 patients entered into the 3-year follow-up phase. During the 3-year follow-up phase 641 (82 %) patients withdrew from the follow-up. The reasons included withdrawal of consent (*n* = 235), lost to follow-up (*n* = 317, death (*n* = 16, 8 = cardiovascular events, 2 = cerebrovascular events, 2 = pneumonia, 3 = suicide, 1 = cancer) patients moving into a different town (*n* = 34), difficulties coming to the study visits (*n* = 23).

Patients remaining in the follow-up sample were significantly more women, had lower rates of personality disorders, were less often referred to inpatient treatment due to suicidality, were more often living in a relationship, were less often discharged against medical advise, were longer hospitalized and had higher HAMD-17 baseline scores (Table 1).

**Table 1:** Differences between patients lost to follow-up (drop-outs) and the completer sample

	Drop-out (*n* = 641)	Completer (*n* =143)	OR	*p*-value	
Female	375 (59 %)	96 (67 %)	1.39	0.017	
Positive family anamnesis	241 (38 %)	59 (41 %)	1.12	0.42	
Recurrent depression	337 (53 %)	84 (59 %)	1.24	0.11	
Any personality disorder	260 (41 %)	44 (31 %)	0.64	0.043	
Suicidality admission reason	337 (53 %)	61 (43 %)	0.66	0.001	
Living with a partner	248 (39 %)	81 (57 %)	2.04	<0.001	
Discharged against medical advise	64 (10 %)	7 (5 %)	0.47	0.009	
Number of hospitalisations before admission	0 ± 2.00 (0–21)	0.5 ± 2.00 (0–15)		0.15	
Inpatient treatment time (days)	49 ± 47.50 (1–278)	56 ± 46.00 (1–363)		0.007	
Age	44.4 ± 12.30 (18.2–69.6)	45.7 ± 11.70 (18.6–65.9)		0.1	
Age at onset	38.1 ± 12.81 (10–69)	38.3 ± 12.61 (9–65)		0.89	
HAMD 17 baseline	22.8 ± 5.88 (2–40)	21.7 ± 6.08 (1–40)		0.005	
HAMD 17 discharge	7.8 ± 5.79 (0–31)	7.7 ± 5.46 (0–30)		0.77	

### Depression ratings

Remission rates, HAMD-17 and MADRS mean scores for observed case analysis and last observation carried forward method are summarized in Table 2.

**Table 2:** MADRS and HAMD total scores (and standard deviation) and proportion of responders (50 % HAMD-17 baseline reduction) and remitters (HAMD-17 ≤ 7) at discharge, year 1, year 2 and year 3: last observation carried forward and completer analysis

	*N*	DISCHARGE	YEAR 1	YEAR 2	YEAR 3
Remission rate (LOCF)		423 (54 %)	431 (55 %)	436(56 %)	451 (58 %)
Remission rate (OC)		92 (64 %)	89 (62 %)	84 (59 %)	99 (69 %)
HAMD-17 (LOCF)		8.8 (±6.8)	7.4 (±7.4)	6.3 (±6.8)	6.6 (±6.8)
HAMD-17 (OC)		7.6 (±5.9)	7.2 (±7.8)	7.1 (±7.4)	6.1 (±6.4)
MADRS (LOCF)		12.5 (±7.6)	10.9 (±9.9)	10.6 (±10.1)	10.6 (±10.1)
MADRS (OC)		11.9 (±6.8)	9.4 (±11.2)	9.3 (±10.8)	8.3 (±9.6)

### Remission

Remission rates at discharge 1- ,2- and 3-year follow-up were 54 %, 55 %, 56 % and 58 %, respectively (LOCF). Completer analysis showed remission rates of 64 %, 62 %, 59 % and 69 % (Table 2).

**Fig. 2:**
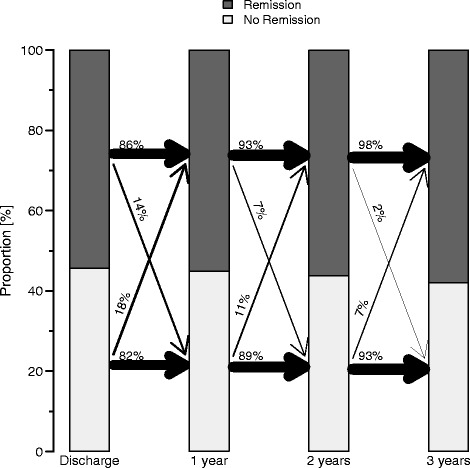
The year to year movement from discharge on until the end of the 3-year follow-up of patients being in HMAD-17 remission (dark) or non-remission (light) for the LOCF sample (*N* = 784). The width of the arrows corresponds to the magnitude of the percentage. For example, from discharge to year one 86 % stayed in remission whereas 82 % stayed non-remitters. 14 % of non-remitted patients became remitters and 18 % of remitted patients lost remission at the 1-year follow-up

According LOCF 86 %, 93 % and 98 % maintained remission after one, two and three years. And only 14 %, 7 % and 2 % switched to non-remission after one, two and three years (Fig. 2).

**Fig. 3:**
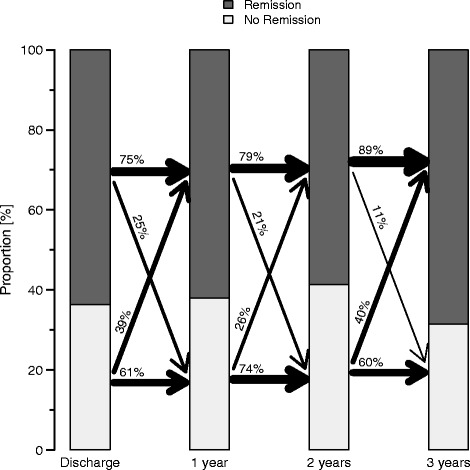
The year to year movement from discharge on until the end of the 3-year follow-up of patients being in HMAD-17 remission (dark) or non-remission (light) for the OC sample (*N* = 143). The width of the arrows corresponds to the magnitude of the percentage. For example, from discharge to year one 75 % stayed in remission whereas 61 % stayed non-remitters. 39 % of non-remitted patients became remitters and 25 % of remitted patients lost remission at the 1-year follow-up

In the completer sample the remission status was constant in 75 %, 79 % and 89 % after one, two and three years. 25 %, 21 % and 11 % switched to non-remission from the preceding to the following year after 1, 2 and 3 years (Fig. 3).

### Illness course

67 % of all discharged patients remitted symptomatically at some time point during the 3-year follow-up and 43 % maintained remission, 33 % never reached a remitted state, whereas 24 % had a fluctuating course (LOCF).

In the completer sample 88 % remitted at some time point during the 3 year period, only 36 % of all patients maintained their remission status throughout all visits, 12 % did never remit and 52 % showed a fluctuating course (OC) (Table 3).

**Table 3:** Total numbers (%) of patients remaining remitters/no remitters/respondents/no respondents after three years: last observation carried forward and completer analysis

	LOCF	OC
Remission	337 (43 %)	51 (36 %)
No Remission	259 (33 %)	17 (12 %)

### Treatment

In order to get an estimate for the development of the prescribed medication and treatment we relied on the observed cases (OC) of 143 patients.

**Fig. 4:**
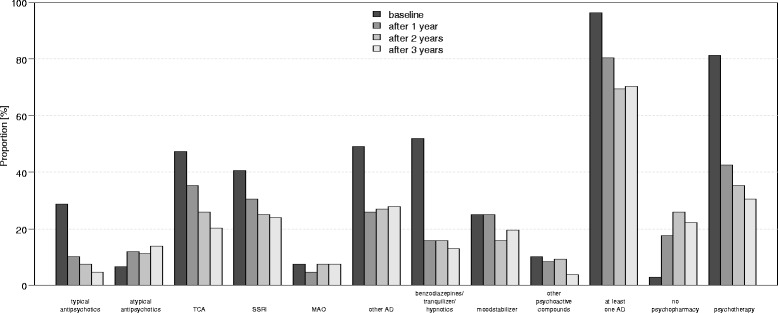
Naturalistic treatment of patients at discharge and after 1, 2 and 3 years of follow-up, the data presented here relates to the 3-year completer sample (OC, *N* = 143)

At discharge 96.3 % of 143 patients had at least one antidepressant, at three year follow-up still 70.4 % of all patients were taking a minimum of one antidepressant, whereas 21.4 % were taking no psychopharmacologic medication at all. Of the 70.4 % patients taking antidepressants, 23.8 % got TCAs, 21.4 % SSRIs and 28 % got dual acting antidepressants (either venlafaxine or mirtazapine) and 6.4 % received MAO inhibitors (Fig. 4).

Apart from atypical antipsychotics (rising form 6.5 % at discharge to 14.9 % at 3-year follow-up) the prescription rates of all other medication classes declined. Benzodiazepines and tranquilizers showed the largest decline (from 51.9 % at discharge to 13.0 % at 3-year follow-up), followed by declining rates of patients receiving psychotherapy (from 81.5 % to 30.6 % at 3-year follow-up).

## Discussion

In this report we applied a descriptive approach and focussed on remission rates and its stability for a series of depressed subjects for a period of 3-years.

### Cumulative remission rates

From that perspective the outcome looks quite promising: 67 % of the LOCF sample and most patients of the OC (88 %) sample recovered at some time point during three years (cumulative remission rate). These cumulative rates are in a comparable range to other naturalistic long-term follow-ups. Holma found 88.5 % after 5 years, O’Leary 88 % after 3 years and Ramana 80 % after 2 years [4, 6, 7]. Also the landmark study by Keller about the naturalistic 5-year course of 431 subjects with major depression found cumulative recovery rates (defined as 8 consecutive weeks with no or minimal symptoms) of 70 % after 1 year, 81 % within 2 years, 87 % within 4 years and 88 % within 5 years [8].

### Absolute response and remission rates

In contrast to cumulative rates, absolute remission rates at a certain follow-up time tend to be considerably lower. With respect to remission rates after one, two and three years, the LOCF analysis revealed 55 %, 56 % and 58 % of remitters in the present study. With respect to observed cases after one, two and three years, almost 62 % completer after one, (59 %) after two and (69 %) after three years met criteria for remission (OC).

The naturalistic Vantaa sample comprised 163 outpatients (OC sample) and applied DSM-IV criteria over a continuous period of two months [4]. After five years 50 % of the observed cases were in full remission in this follow-up study.

The MADRS remission rates of observed cases (MADRS < 9) of the naturalistic SLICE study on a primary care population (*n* = 1031) after 1- (70.7 %) and 2-years (75.3 %) were higher than in the present report [22]. But it needs to be considered, that a primary outpatient sample usually includes less severe and less refractory patients in comparison to a tertiary care inpatient population as in the report at hand [23].

The PROSPECT study reported HAMD-17 remission rates of the observed cases of an intervention group receiving algorithm-based interventions of 40.1 % after one and of 49.7 % after two years although the mean HAMD baseline severity (18.1 vs. 24), was only in a moderate range [24].

Thus, all in all the rates of the present report are in a similar range to other naturalistic data with a tendency towards the upper range, despite its tertiary referral infrastructure. An important limitation in that context is the high drop-out rate of 82 % leading to a selection of patients with favourable outcomes (also see limitations).

Although “real world” patients and patients included in randomized controlled trials are not easily comparable [25, 26], a look into long-term data of a recent randomized controlled trial, might still be informative. For example in the Co-Med trial remission rates after 7-months were lower with 48 % (LOCF). But here the observational period was shorter and the remission criterion stricter (patients had to be in a remitted state on 2 consecutive visits) [27]. Another example is the PREVENT trial, here the remission rates of this double blind randomized controlled long term trial comparing fluoxetine against venlafaxine (*N* = 268) were higher at year one (67–68 %) and (71 %–77 %) at year two (OC) [28].

### Stability of remission and illness course

The stability of remission for LOCF and OC analysis is illustrated in flow chart diagrams (see Figs. 2 and 3). Due to the high drop-out rate, the OC analysis seems to be the most reliable one in that respect (see limitations).

The highly fluctuating course of major depressive disorder is reflected by 52 % of patients (OC) showing a fluctuation from remission to non-remission and vices versa throughout the three years. This result is in line to the previously published corresponding relapse rates which have been retrospectively assessed at each follow-up. Of the 458 patients 155 (33.6 %) experienced at least one severe relapse during the 3-year follow-up period. The highest rate was found in the first month and the first year (25.3 %) after discharge from inpatient treatment declining to 16.1 % two years thereafter [10].

A finer grained picture can be obtained by only looking at the switch rates from remission to non-remission. These rates vary between 11 % and 25 % with 45 % loosing remission at some time point during the three years. Only 36 % of all patients (and 55 % of initially remitted patients) stayed in remission during the whole time (OC). The recent naturalistic PREDICT-NL study followed 174 primary care outpatients with major depressive disorder (out of 1338 attendants) for a period of three years. In line with our results the authors found a rate of 40 % with a fluctuating course and rate of 43 % staying in remission right from the start. The benign rates can again be well explained by the milder and less complex cases of a primary care outpatient population. However, still 17 % of patients in the PREDICT-NL study had a chronic course and stayed in an episode for the whole 3 years [26]. In the report at hand, 12 % of all discharged patients stayed in non-remission from discharge up to 3-years and thus had a chronic course (OC, Table 3). This level of chronicity is also in accordance with earlier reports. Keller reported that 12 % patients of the CDS study did not reach recovery after 5 years [9]. Likewise Jules Angst reported in his 21 year follow-up of 406 initially hospitalized patients that 13 % of all patients developed a chronic course [27]. Spijker found a slightly higher rate of 20 % of patients with MDD who had not fully recovered after a period of two years in the NEMESIS study [29].

### Treatment

After three years 70.4 % of the completer sample received at least one antidepressant. A recent systematic review including 14 large observational naturalistic/epidemiologic surveys reported adherence rates ranging from 30–97 % (median 67 %) [30]. The comparably high psychotherapy rate as well as the high rate of patients being in specific mental health care (84 % year one, 81 % year two and 78 % year three), together with a higher chance of adherent patients staying in the completer sample might have led to high adherence rates in the completer sample. In addition it should be kept in mind that, the German health care system provides healthcare insurance for all community members, allowing a free choice of doctor. Therefore, the encouraging adherence rates can partly be traced back to specialized mental health care advanced by the German insurance policy.

### Limitations

The most important limitation pertains to the high dropout rate. Attrition rates in long-term studies of similar time spans, range from 18 % [31], to 72 % [32, 33]. The Texas algorithm project showed similar attrition rate after one year of 47 % [34]. Thus, the three-year attrition rate of the present study of about 82 % of the patients entering the follow-up appears to be within a high range.

Amongst others, one reason for the high attrition rate in the study at hand may have been due to the way participants during the follow-up had to be contacted in accordance with the study protocol. Participants were only allowed to be contacted via letters and not via telephone or email. Thus, over the three years twenty mailing waves have been performed. In addition, no telephone interviews were intended for the yearly visits but face to face interviews had to be performed instead. Although these interviews might result on the one hand in a higher data quality, they may led to higher attrition rates on the other hand. For future studies contact via Email and especially telephone calls, which have shown to increase retention time in trials are clearly preferable [35].

The drop out analysis revealed that patients staying up to three years in the follow up exhibited variables that are associated with better longterm outcome (female gender, less comocrbid personality disorders, living more often with partner, less often discharged against medical advice, longer inpatient treatment time, lower Hamilton baseline score, (Table 1)).

These variables are largely in line with the variables associated with lower attrition in the STAR*D Study and the Texas algorithm project [34]. Patients of the drop out sample showed significantly lower remission rates in step one and two of STAR*D [34, 36]. Thus the outcome of the OC sample of the report at hand are if anything optimistic and it seems likely that we have overestimated positive outcome[37, 38].

Nevertheless, this sample of 3-year long-term data of initially hospitalized, naturalistic treated and extensively reevaluated patients with major depression is still one of the largest European follow-ups.

Since there were broad inclusion and only few exclusion criteria, patients who would have been excluded in most randomized controlled trials, were included in our study. This is a limitation and strength at the same time. The results of this study could be more generalizable to routine clinical practice and exert high external validity for adult inpatients with major depressive disorder. On the other hand internal validity is reduced due to the lack of any control group. Therefore conclusions regarding treatment effects are very limited. In addition these results might not be easily generizable to outpatient populations or elderly patients [39].

We also strictly focused on remission on certain points in time without applying any duration thresholds. Several authors suggested that a patient should at least remain in remission for at least 8 consecutive weeks before he can be considered as recovered [2, 9]. Moreover, since there was no course interview implemented, we cannot rule out that a patient who is in remission at all three visits may have experienced episodes or more illness activity in between the visits. Thus our rates of remission are, if anything, optimistic. Modern outcome measures like ecological momentary assessment techniques (EMA) could provide promising tools which might complement such traditional outcomes measures.

Thus, in summary the results of the 3-Year completer sample, even if positively biased, are rather disappointing with 12 % showing a chronic course, 52 % a highly fluctuating course and only 36 % percent a stable remission and call for future strategies enhancing long-term outcome.

### A comment on missing data in long-term trials - Last observation carried forward (LOCF) and observed cases (OC)

Valid analyses of longitudinal data are complex and difficult, especially if data are missing for reasons that are related to the outcome. We used two traditional methods to address this problem: 1) the LOCF method which imputes data by carrying the last observation forward and 2) the completer or observed case analysis by only including those patients who had an observation at each visit and at endpoint. It is often argued that the bias in LOCF leads to a “conservative” (under-) estimation of treatment effects. On the other hand, the completer analysis or observed case analysis is assumed to lead to an overestimation of treatment effects. Therefore a combined approach can help to get a realistic idea of the outcome rates.

In the present study LOCF indeed estimated absolute remission at the predefined time points rates more conservatively but overestimated the stability over the long term course due to the high rate of patients lost to follow-up. For example stable remitters were higher in the LOCF analysis than with the OC method (43 %, LOCF vs 36 % OC). In contrast, the observed case analysis showed less conservative absolute outcomes (e.g. yielding higher remission rates after 1, 2 and 3 years) but allows a more realistic look on the development over the long term course.

In recent years modern imputation methods like mixed models for repeated measures are becoming more and more common and are often recommended as preferable method. On the other hand it has been emphasized that they are not the cure for all problems associated with missing data [40, 41].

## Conclusion

The present results may reflect clinical wisdom and experience that patients with major depression have an inherent tendency to recover and that we can reassure our patients that in the long run almost all patients will experience a significant symptom reduction. But on the other side a significant percentage will again become more symptomatic in the long run. Thus major depressive illness appears to have a highly unstable and fluctuating course. Furthermore, 12 % of patients did not remit initially and did not reach remission by the end of the study. The persistence of significant levels of psychopathology is associated with significant psychosocial impairment and calls for long-term treatment intervention studies addressing this unmet need.

### Ethics

All methods were described in detail in a study protocol, which allowed post-hoc analyses. Signed written informed consent was obtained from all participants.

The respective local Ethics Review Committee of each participating site has approved the study protocol as follows:

The Ethics Review Committee of the Ludwig Maximillian’s University of Munich has approved the protocol for the sites: LMU, MPI, Inn-Salzach-Clinic and Isar-Amper-Clinic;

The Ethics Review Committee of the Campus Charité Mitte in Berlin has approved the protocol for the sites: Auguste-Viktoria-Hospital, St Joseph-Hospital, St Hedwig-Hospital and Campus Benjamin Franklin.

The Ethics Review Committee of the University of Düsseldorf approved the protocol for the site Düsseldorf.

The Ethics Review Committee of the University of Halle has approved the protocol for the site Halle.

The Ethics Review Committee of the University of Heidelberg approved the protocol for the site Heidelberg.

### Consent to publish

Non-Applicable.

### Availability of data and material

The data will not be made available in order to protect the participants identity.
